# A "Revolting" Crime in Scotland

**Published:** 1921-05-14

**Authors:** 


### A "Revolting" Crime in Scotland.

By way of the *New Witness*, a shocking crime against children is reported from Scotland. According to our agonised contemporary, a congress of school medical officers at Edinburgh laid down the principle that physical fitness is the foundation of all usefulness in the schools, "which, of course, is sheer animalism." What is worse, the Scottish Board of Health was present and endorsed this view, which leads the distressed writer in the *New Witness* to remark that "since the declared purpose of the Board this year is to supervise in a special way the health of the children at school, it will be interesting, *though revolting* [our italics], to watch their attacks on the little ones." As if this were not enough, Professor Dreyer, of Oxford, spoke at the same gathering "with apparent seriousness of the need of examining the trunk, length, and chest measurements of the children, with a view to ascertaining their vital capacity." "What on earth," asks our horrified contemporary, as far as it is able to articulate at all, "will these doctors be up to next? To the great chagrin of the teachers, the schools are being converted into surgeries, and the children into subjects for experimentation. Education as we once knew it is going." And *Punch*, as the club bores have said for a generation, is notoriously not what it used to be.

It is as difficult to understand what the *New Witness* really wants as to appreciate the meaning of its objection to the modern ideal of physical fitness in the schools. Does its editor really imagine it is "faddism" to apply in practice the theory that a healthy child is likely to be better taught, to grow into a much more intelligent citizen, than an unhealthy one? Is it "inane" to try to find

**THE PUBLIC WELL-BEING**—(*continued*).

out what is a child's potential efficiency before you begin to educate the child? Who can have told him that the teachers are "chagrined" because their work is being simplified, or that the school clinic—one of the best life-saving and disease-preventing institutions in the country—is a place where children are "subjects for experimentation"? The testimony of the *New Witness* appears to be about half a century old, and to be as irrelevant as it is anachronistic. We do not quote its views because we fear that they will have any weight with the Scottish Board of Health, or because they have any intrinsic importance. No very great attention is usually paid to a man who argues that the earth is flat, or that a wooden sailing ship moves faster in the water than a modern torpedo-boat destroyer. But that a serious newspaper should publicly express itself in the manner described shows that there is still a very real need in unexpected quarters for education—not "as we once knew it," but quite up to date—in questions of public health.

